# Premature farrowing and stillbirths in two organic sow farms due to riboflavin deficiency

**DOI:** 10.1186/s40813-023-00308-y

**Published:** 2023-05-05

**Authors:** Camilla Torreggiani, Dominiek Maes, Luigi Franchi, Valeria Raffi, Elena Borri, Alice Prosperi, Chiara Chiapponi, Andrea Luppi

**Affiliations:** 1 IZSLER, Istituto Zooprofilattico Sperimentale Della Lombardia E Dell’Emilia-Romagna, Parma, Italy; 2 Veterinary Practitioner, Progeo, Italy; 3 Unit of Porcine Health Management, Faculty of Veterinary Medicine, Ghent University, Ghent, Belgium

**Keywords:** Vitamin B2, Stillbirth, Weak-born piglets, Organic farms

## Abstract

**Background:**

Riboflavin deficiency can lead to premature farrowing, stillborn piglets, weak-born piglets and neonatal death. Riboflavin (vitamin B2) is considered essential for reproductive function. The longer the period on riboflavin-deficient diets, the more severe the clinical signs become. Litter size as well as body size of piglets can also be considered risk factors that may contribute to the problem.

**Case presentation:**

This case report involved two organic farms of 320 (farm A) and 250 sows (farm B). Between 2019 and 2020, premature farrowing with weak-born or stillborn piglets and severe intra-litter mortality, ranging from 60 to 100% were observed. Investigations for infectious causes of reproductive disease, drinking water quality and general feed composition were performed, but showed no significant results. Feed composition was subsequently evaluated more in detail. Riboflavin levels were very low specifically 1.25 mg/kg of diet (3.75 mg/kg of diet is the NRC minimum recommended level). Riboflavin as a vitamin complex supplement (B complex) was administered to sows one month before the farrowing date and this led to a rapid improvement of the problem such that no stillbirth or intra-litter mortality was observed.

**Conclusions:**

The clinical presentation, the low riboflavin levels in the feed below the recommended levels for gestating sows and the effectiveness of the riboflavin supplementation, led to an *ex juvantibus* diagnosis of this deficiency condition. This case report highlights that riboflavin deficiency during gestation should be considered in case of premature parturition and stillborn litters.

## Background

Perinatal piglet mortality is a multifactorial problem leading to major economic and animal welfare problems in pig farms around the world. Stillborn piglets may be caused by infections and be associated with many non-infectious risk factors. The risk factors can be further categorized as genetic, maternal, piglet and environmental factors, with a possibility of interaction between them [1]. Many infectious pathogens can cause reproductive problems and it is also well known that farrowing management plays an important role in stillbirths, for instance, birth induction, prolonged farrowing, dystocia and inappropriate use of uterotonics.

Increasing litter size is a risk factor for stillbirths [2–6] and litter sizes of greater than 12 piglets showed a higher risk of stillbirth [7]. Maternal nutrition can directly impact foetal development and, therefore, poor maternal nutrition or imbalances in the diet are significant risk factors. Nutritional supplementation during late gestation is a strategy for improving piglet survivability [8].

Riboflavin, also known as vitamin B2, is considered essential for reproductive function. In pregnant sows, riboflavin deficiency can lead to premature births, stillbirths, and death [9]. It is noteworthy that only a few feed ingredients commonly used for pigs offer a sufficient supply of riboflavin to meet the nutritional requirement. Swine diets that are largely based on grains are predisposed to being deficient in riboflavin. Slower growth rates and feed efficiency are common signs in many animal species [10].

In fact, riboflavin is required as part of many enzymes essential to the utilisation of carbohydrates, fat, and protein. More than 100 enzymes are known to bind flavin adenine dinucleotide (FAD) or flavin mononucleotide (FMN) in animal and microbial systems. Riboflavin and related natural flavins participate in numerous and diverse reactions, perhaps more than any other vitamin-coenzyme group.

Riboflavin functions in flavoprotein-enzyme systems to help regulate cellular metabolism, and is also specifically involved in the metabolism of carbohydrates. Riboflavin is also an essential factor in amino acid metabolism as part of amino acid oxidases [10].

This case report describes an episode of increased premature birth and stillbirth rate in two organic pig farms. This case shows that riboflavin deficiency in pregnant sows should be considered in the differential diagnosis when such reproductive problems occur.

## Case presentation

The case report involved two organic farms of 320 (farm A) and 250 sows (farm B), both practising a three-week batch production. The genetics were Topigs 20 and 60, with Topigs Fomeva11 terminal boar and with in-house production of great grandparent (GGP) and grandparent (GP) gilts. In both farms, the same organic pelleted sow feed was used. Sows were fed individually with electronic sow feeders during gestation and lactation. Lactating diet started about a month before entering the farrowing room. They were fed ad libitum in the farrowing room. Body condition scores (BCS) were evaluated by measuring backfat and by weighing the sows. The scores were always within reference levels. Sows were housed in farrowing pens, each pen measured 7.5 square metres and also had an external box of 2.5 square metres with a solid floor, and straw as a bedding material. The ambient temperature was 21–22 °C, while the nest for the piglets was kept at 26–27 °C. Sows were moved from the gestation unit to the farrowing unit five days prior the expected farrowing day. As the farms were organic, birth induction was not allowed and the use of uterotonics was only possible in specific sporadic cases in order to guarantee animal welfare. Farrowing management was not modified before or after the onset of the problem, while the length of gestation remained the same for the whole period of time.

Breeding animals (gilts and sows) were vaccinated against Suid Herpesvirus 1 (every 3 months), Parvovirus, Leptospira and Erysipelas (every 4 months) and gilts were vaccinated against neonatal colibacillosis and clostridiosis some weeks prior to first farrowing. The farms were infected with porcine reproductive and respiratory syndrome (PRRS), but there were no clinical problems.

In farm A sows had a litter size that ranged from 15 to 23 with an average litter size of 19 piglets per litter (total born piglets), 89.6% of piglets were live-born while the stillborn rate was 10.4%, the pre-weaning mortality was around 18% and the number of weaned piglets per sow per year was 28. The average gestation length was 114.5 days and the return to oestrus was around 15% (Table 1).

**Table 1:** FARM A. Reproductive performance before, during and after the occurrence of riboflavin (vit B2) deficiency (during the monitoring period of 18 months, from January 2021 to June 2022) following treatment

Parameter	Before vit. B2 deficiency	During 2020	Monitoring period
Average litter size (Total born piglets)	19	18.9	18.8
% of live born piglets	89.6	85.0	89.5
% of stillborn piglets	10.4	15.0	10.5
Pre-weaning piglet mortality (%)	18	50	20
Number of weaned piglets per sow/year	28	16	27
% of sows showing return to oestrus	15	17	15

As farm B was a newly established farm, there was no historical data about reproductive performances.

Premature farrowing and severe intra-litter mortality were observed in both farms from October 2019 until December 2020. The problem was distributed all year around and no seasonal effect was observed. The percentage of animals affected ranged from 60 to 100% and included first parity as well as multiparous sows; primiparous sows showed the problem earlier compared to multiparous sows, even though no differences have been observed from a clinical point of view (Table 2).

**Table 2:** FARM B. Reproductive performance before, during and after the occurrence of riboflavin (vit B2) deficiency (during the monitoring period of 18 months, from January 2021 to June 2022) following treatment

Parameter	Before vit. B2 deficiency*	During 2020	Monitoring period
Average litter size (Total born piglets)	–	16.1	17.3
% of live born	–	88%	87%
% of stillborn piglets	–	12%	13%
Pre-weaning piglet mortality (%)	–	40%	20%
Number of weaned piglets per sow/year	–	17	24.12
% of sows showing return to oestrus	–	20%	18%

*No historical data about reproductive performances. Farm B was a newly established farm

In farm A, first parity sows (approximately 20% of the total sows), presented premature farrowing during October 2019. This occurred 3–4 days before the expected date of farrowing, and resulted in weak-born or stillborn piglets, in particular stillbirth type II. To control premature farrowing, from November to the end of December 2019, progestogen (Regumate 5 ml/sow) was administered at day 110, 111 and 112 of gestation. This treatment decreased the number of sows with premature farrowing, but the incidence of weak-born or stillborn piglets remained the same. As soon as the administration of progestogens was suspended, premature births were once again observed. During 2020, the situation worsened with premature farrowing and high piglet mortality 15–24 h after birth, and intra-litter mortality ranging from 60 to 100%. The total number of piglets born remained the same, that is between 15 and 23 piglets per litter. Subsequently, approximately 20% of the multiparous sows showed similar problems. In farm B, a newly established herd, a high incidence (48%) of premature farrowing in first parity sows (3–4 days before the due date of delivery) was observed in March 2020. This was systematically observed in subsequent batches; more specifically 30–40% of the sows exhibited premature farrowing. During August and September 2020, the second parity sows experienced the same problems.

In both farms, the problem persisted throughout 2020 and led to the loss of 1600 (farm A) and 970 (farm B) piglets respectively. The sows were not clinically diseased and did not show other fertility problems such as repeat breeding or abortions.

Between 2019 and 2020, 88 piglets from farm A and 117 piglets from farm B were sent to the Diagnostic Laboratory of Parma at Istituto Zooprofilattico Sperimentale of Lombardia and Emilia-Romagna for post-mortem examination and laboratory investigations. Necropsies were conducted on stillbirth piglets, weak born piglets and also on piglets that died during lactation. At necropsy, piglets did not show significant gross lesions. Bacteriological examination performed for each piglet on samples of liver, kidney, spleen and brain did not show pathogenic bacteria.

Investigations for PRRSV, Virotype PRRS RT-PCR Kit (Kit Indical), PCV2 [11], Parvovirus (PPV) [12], Encephalomyocarditis virus (EMCV) [13], Porcine Cytomegalovirus (PCMV), Atypical Porcine Pestivirus (APPV) [14], Sapelovirus (PSV) [15], Porcine Circovirus type 3 (PCV3) [16], *Chlamydia* spp. [17], *Leptospira* spp. [18], *Brucella* spp. [19] and *Toxoplasma gondii* [20] were performed on different samples from both farms (Table 3).

**Table 3:** Analyses of different pathogens, sample type and methods used on the piglets from both farms that were submitted to the laboratory

Etiological agents	Number of samples taken	Sample type	Methods	References
APPV	9	Pooled internal organs (brain, lungs, heart, kidney, spleen, lymph nodes)	RT-PCR	Schwarz et al. [14]
EMCV	13	Heart	RT-PCR	Bakkali Kassimi et al. [13]
PCV2	69	Heart	qPCR real time	Olvera et al. [11]
PCV3	32	Heart	PCR real time	Palinski et al. [16]
PCMV	7	Pooled internal organs (spleen, tonsils)	PCR	artus® CMV RG PCR Kit Qiagen
PPV	60	Liver	PCR	Kim et al. [12]
PRRSV	120	Spleen, lungs	RT-PCR real time	Virotype PRRS RT-PCR Kit (Kit Indical)
PSV	5	Pooled internal organs (liver, spleen, lungs, intestines)	RT-PCR	La Rosa et al. [15]
*Brucella* spp.	25	Pooled internal organs (spleen, uterus, testicles)	PCR real time	Bogdanovich et al. [19]
*Chlamydia* spp.	25	Pooled internal organs (spleen, liver, lungs)	PCR real time	Ehricht et al. [17]
*Leptospira* spp.	25	Pooled internal organs (lungs, liver, kidney)	PCR real time	Bedir et al. [18]
*Toxoplasma gondii*	24	Brain, pooled internal organs (lungs, heart, liver, kidney, spleen, brain)	PCR real time	Menotti et al. [20]

Five pooled samples belonging to 3 different litters from farm A were positive for PCV3. The cycle quantity (Cq) values were 18.3, 37.2, 32.3 and 19.2. Cq values higher than 38 were considered negative. Two pools of piglets belonging to the same litter from farm B tested positive for PSV.

Samples from brain, cerebellum, kidney, spleen, liver, lungs, heart, and intestines were collected during necropsies from all cases and fixed in 10% neutral buffered formalin and a sample of them were submitted for histopathological evaluation following standardised methods. Samples were submitted for histopathological evaluation to confirm the results of PCRs (tissues belonging to all piglets of pools resulted positive for PCV3 in farm A and PSV in farm B) or to investigate possible microscopic lesions in samples that were PCR negative. No significant histopathological lesions were found in any of the samples.

The feed administered to sows in both farms was tested to assess levels of zearalenone, using an ELISA test. The levels were very low, namely between 36 and 84 ppb, and not considered to be as clinically relevant [21].

The drinking water in both farms (ground water) was tested for chemical, physical and microbiological parameters. The results of these investigations did not show any abnormalities. In the absence of reference values for livestock, we took into consideration reference values for human consumption (Table 4).

**Table 4:** Drinking water analysis

Exams	Methods	Unit of measurement	Results	Reference D.L. 31 02/2001 [22]
*Chimical parameters*				
Colour			Absent	Absent
Odour			Absent	Absent
PH	Potentiometric		7.48	6.5–8.5
Electrical conductivity	Potentiometric	µS/cm^−1^	543	
Dry residue	Gravimetric		383	< 1500
Ammonia	Indophenol	mg/l	< 0.1	< 0.5
Nitrite	Griess	mg/l	< 0.02	< 0.1
Nitrate	Photometric	mg/l	1.4	< 50
Chloride	Mohr	mg/l	11	< 200
Fe^2+^	Photometric	mg/l	1.018	< 0.2
Manganesium	Photometric	mg/l	0.172	< 0.05
Hardness	Eriochrome black titration	°F	27.2	15–50
Alkalinity	Methyl orange titration	mg/l	310	
Organic matter	Kubel	mg/l O_2_	0.6	< 5.0
Phosphorus	Irsa	mg/l	< 0.3	< 5.0
Turbidity	Turbidimeter	U.J	2.5	< 4.0
*Bacteriological parameter*				
Total bacterial count	PCA inclusion	u.f.c./ml	36	
Total coliform	Endo agar (microfiltration)	u.f.c./100 ml	Absent	Absent
*Escherichia coli*	Endo agar (microfiltration)	u.f.c./100 ml	Absent	Absent
*Enterococcus* spp.	Aesculin (microfiltration)	u.f.c./100 ml	Absent	Absent

A total of 50 samples, from both farms, from gestating and lactating sows, were collected for feed evaluation using High Performance Liquid Chromatography Diode Array Detector (HPLC–DAD) methods. These 50 samples were collected from different silos and stocking units, and at different timepoints from the end of 2019 throughout 2020. The analysis results showed nutrient levels above the National Research Council (NRC) minimum recommended values, including the levels of different vitamins, with the exception of riboflavin, for which the detected level was 1.25 mg/kg of diet (Tables 5 and 6). Considering that the recommended level for gestating sows is 3.75 mg/kg of diet (7.87 mg/day) [23], the riboflavin level in our sample was 3 times lower. Therefore, riboflavin deficiency was considered to be a possible the cause of the problems. Riboflavin as a vitamin complex supplement (B complex) (Bicomplex Vet 250 ml, Izo, 10 ml/sow, corresponding to 30 mg of riboflavin, IM or per os) was administered intramuscularly once per day for 3 consecutive days one month before expected farrowing, and subsequently, daily *per os*, for 30 days until farrowing (Tables 7 and 8). In order to evaluate the effectiveness of the treatment, the practitioners administered vitamin B2 close to parturition, approximately 15 days before the due date.

**Table 5:** Feed composition for gestating and lactating sows in both farms

Ingredients	Feed composition % for gestating sows	Feed composition % for lactating sows	Reference values for gestating Topigs sows [24]	Reference values for lactating Topigs sows [24]
Moisture	12.70	12.80	11.50–13.20	11.50–13.20
Crude protein	14.50	16.50	13–14	16–18
Crude fat	3.60	5.00	3–4	5–6
Crude fiber	6.10	5.30	6–8	Min. 5
Crude ash	5.50	5.80	5–6	5–6
Calcium	1.22	1.23	0.65–0.85	0.98–1.05
Digestive phosphorus	0.35	0.4	0.22–0.30	0.3–0.4
Sodium	0.20	0.20	0.15–0.2	0.18–0.20
Lysine	0.60	0.85	0.45–0.88	0.85–0.95

**Table 6:** Average levels of vitamins detected in the feed of gestating sows, and the minimum requirements suggested by the National Research Council (NRC) and for Topigs sows [23, 24]

Vitamin	Unit of measurement	Levels in gestating sow feed N = 29	Levels according to NRC requirements [23]	Levels according to Topigs sow feed [24]
A	UI/kg of diet	9600	4000	10,000
D3		1600	800	2000
E		80	44	40
K3	mg/kg of diet	3.60	0.50	1
B1		1.60	1.00	1
B2		1.25	3.75	4
B6		2.00	1.00	1
B12		0.040	0.015	0.03

**Table 7:** Vitamin complex composition (Bicomplex Vet 250 ml, Izo)

Vitamin complex composition	mg/ml
Vitamin B1	5.00
Vitamin B2	3.00
Vitamin PP	20.00
Vitamin B5	12.00
Vitamin B6	2.00
Vitamin B12	0.002

**Table 8:** Premix vitamin complex (Bicomplex_tech)

Premix vitamin composition	mg/kg
Vitamin B1	24,000
Vitamin B2	1300
Vitamin B6	600
Vitamin B12	6
Niacinamide	4000
Cholin	50,000
Calcium	2.000
Amino acids, DL-methionine	25.000

This supplementation led to the resolution of the problem, and no problems with premature farrowing or increased stillbirths were observed during a monitoring period, specifically 18 months, from January 2021 to June 2022.

## Discussion and conclusions

This clinical case described a severe outbreak characterised by premature farrowing and weak-born and/or stillborn piglets in two organic sow farms. The screening for different pathogens to be considered in the differential diagnosis in case of reproductive problems in sows demonstrated sporadic positivity to PCV3 (farm A) and PSV (farm B).

PCV3 was first described in 2015 [16] and, since then, its role in different diseases has been debated. Despite the worldwide distribution of PCV3, there is little evidence of PCV3 detection within lesions of diseased animals [25]. Saporiti et al. [25] proposed diagnostic criteria for the individual case definition of PCV3-reproductive disease: (A) stillborn piglet from a litter with a late reproductive problem charactesized by an increased percentage of stillborn and weak-born piglets, (B) mild-to-moderate mononuclear inflammatory infiltrates in the arterial wall of the foetal spleen and (C) moderate to high amount of PCV3 nucleic acid in the damaged arterial area [25]. The most convincing evidence of disease association is that which demonstrates late abortion, malformations, mummified foetuses, stillborn foetuses, weak-born piglets linked to multisystemic lymphoplasmacytic, lymphohistiocytic perivascular inflammation and the presence of viral nucleic acid within these lesions [16].

PSV has been associated with several diseases, primarily gastrointestinal, neurologic, reproductive, and respiratory disorders, but also with subclinical infections. However, for most serotypes, proof of a causal relationship between viral infection and clinical signs is still lacking [26]. The presence of a PSV genome in a sick animal does not imply that this virus is the cause of the clinical signs or lesions. The main lesions caused by a neuro-invasive PSV in infected pigs was characterised by encephalomyelitis [26], while experimentally infected piglets exhibited encephalitis, nonsuppurative myelitis and pneumonia [27]. In our case, the detection of PSV and PCV3 by PCR was not confirmed by the demonstration of diagnostic histopathological findings in piglet tissues. These findings ruled out PSV and PCV3 infections as clinically relevant in this case.

In addition to the investigations for infectious problems, attention was paid to the investigation of non-infectious causes such as zearalenone and to assess drinking water quality and feed ion. As reported above, the nutrient levels were within the NRC recommended ranges, with the exception of riboflavin, for which the levels were three times lower than those recommended by the NRC.

Animals and humans are unable to synthesise riboflavin within tissues and, therefore requirements should be basically met primarily by dietary sources, along with some intestinal microbial synthesis. Riboflavin plays many essential roles in the release of food energy and the assimilation of nutrients, so it is easy to understand why deficiency is reflected in a wide variety of signs among animal species. A decreased growth rate and lowered feed efficiency are common signs in all affected species [10]. Riboflavin is one of the vitamins most likely to be deficient in swine. Swine diets are often based on grains and plant protein sources, such as soybean meal, which are generally deficient in riboflavin. Only a few feedstuffs fed to swine contain enough riboflavin to meet the requirements for growth and reproduction. There are few publications regarding riboflavin deficiency in pigs, but some authors managed to make a list of clinical signs that can affect gilts if they are fed a riboflavin-deficient diet during gestation and lactation. An old study reported that riboflavin deficiency resulted in early parturition, the birth of deformed stillborns, neonatal death, and piglets deficient in riboflavin [4]. Cunha [28] reported that clinical signs for gilts fed with a riboflavin-deficient diet were mainly characterised by complete a loss of appetite and parturition four to 16 days prematurely. The longer the period on riboflavin-deficient diets, the more severe the deficiency signs became. Lower piglet birth weights as well as larger litter sizes are reported as risk factors that may contribute to stillbirth and early piglet mortality both in conventional and organic indoor production systems [2, 3, 29]. In both farms, sows were hyperprolific, with an average litter size of 19 piglets, and this might have worsened the problem, considering that a litter of more than 12 piglets has been mentioned as a risk factor for stillbirths [7].

Riboflavin requirements vary with growth, environment, age, activity, health, other dietary components and synthesis by the host. Only a few old studies have reported the riboflavin requirement in pigs. As an example, Hughes proposed in 1940 that the daily minimum requirement of riboflavin for young growing pigs ranged between 2.2 and 6.6 mg per 100 kg body weight [30]. Krider et al. [31] stated that 3.1 mg of riboflavin per kg of diet represented the practical minimum requirement for weanling pigs fed in dry lot.

More recently, the NRC reported that pigs have a riboflavin requirement of between 2 and 4 mg/kg of diet [32] and as reported above, the riboflavin requirement for gestating sows is 3.75 mg/kg of diet [23]. In general terms, the requirement declines as the pig grows, from 4 mg/kg of feed for pigs with a body weight of 1–5 kg to 2 mg/kg for growing-finishing pigs weighing 50–100 kg [32]. Based upon sow farrowing performance and erythrocyte glutathione reductase (an indicator of riboflavin status), Frank et al. [5] estimated the available riboflavin requirement during gestation to be about 6.5 mg/day. This value was later updated by NRC to 7.87 mg/day [23]. Pettigrew et al. [33], however, observed that 60 mg/day of riboflavin produced a higher farrowing rate than 10 mg/day when these levels were fed from breeding to day 21 of gestation. Using the same criteria, the suggested daily requirement during lactation was about 16 mg/sow [9]. Frank et al. [9] also suggested that first-litter gilts have a higher riboflavin requirement than second-litter sows based on needs for both maternal growth and reproduction.

Zintzen et al. [34] reported that gilts fed a riboflavin deficient diet had a progressively longer average time interval between consecutive oestrus periods, but this was not observed in this case.

An important information that is missing is the evaluation of erythrocyte glutathione reductase activity coefficient (EGRAC), a sensitive biochemical indicator of riboflavin deficiency. Frank et al. [9] reported that to obtain a metabolic index of riboflavin status, sows blood samples collected at day 1 and 24 postpartum, may be used for EGRAC assessment. Unfortunately, once exams showed low riboflavin levels in the feed and we already suspected vit. B2 deficiency, considering the amount of losses for the farmer, the practitioner proceeded to administer vit. B2 to solve the problem, and at that time there were no sows available for sampling.

The clinical presentation, the results of laboratory investigations that ruled out infectious agents, the low level of vitamin B2 in the feed which was far below the NRC’s minimum values, and the effectiveness of the multivitamin supplementation led to an ex juvantibus diagnosis of this deficiency condition. The treatment administered to sows proved to be successful that it could be suggested as a protocol.

This case report highlights that riboflavin deficiency during gestation should be considered in cases of premature parturition and stillborn litters.

## Data Availability

Not applicable.

## Abbreviations

APPV: Atypical porcine pestivirus
Cq: Cycle quantity
EGRAC: Erythrocyte glutathione reductase activity coefficient
EMCV: Encephalomyocarditis virus
ETEC: Enterotoxigenic *Escherichia coli*
FAD: Flavin adenine dinucleotide
FMN: Flavin mononucleotide
GGP: Great grandparent line
GP: Grandparent line
HPLC–DAD: High performance liquid chromatography–diode array detector
PCMV: Porcine cytomegalovirus
PCR: Polymerase chain reaction
PCV2: Porcine circovirus type 2
PCV3: Porcine circovirus type 3
PPV: Porcine parvovirus
PRRS: Porcine reproductive and respiratory syndrome
PRRSV: Porcine reproductive and respiratory syndrome virus
PSV: Porcine sapelovirus
qPCR: Quantitative polymerase chain reaction
